# Very Early Colorectal Anastomotic Leakage within 5 Post-operative Days: a More Severe Subtype Needs Relaparatomy

**DOI:** 10.1038/srep39936

**Published:** 2017-01-13

**Authors:** Yi-Wei Li, Peng Lian, Ben Huang, Hong-Tu Zheng, Ming-He Wang, Wei-Lie Gu, Xin-Xiang Li, Ye Xu, San-Jun Cai

**Affiliations:** 1 Department of Colorectal Surgery, Fudan University Shanghai Cancer Center, Shanghai 200032, China; 2 Department of Oncology, Shanghai Medical College, Fudan University, Shanghai 200032, China

## Abstract

Early anastomotic leakage (AL), usually defined as leakage within 30 post-operative days, represents a severe entity. However, mounting evidence has indicated that majorities of leakage occur within one week after surgery, making late AL rarity. Here we analyzed 101 consecutive colorectal AL, all of which occurred within 30 post-operative days, during Jan 2013 and Dec 2015 in cancer hospital of Fudan University. AL occurring within 5 post-operative days was defined as very early AL (vE-AL). We evaluated risk factors of vE-AL compared with non-vEAL and correlated with post-leakage peritonitis and need of relaparatomy. We found that AL occurred at median time of 7 days after surgery. 23 cases were vE-AL. Reconstruction of post-peritoneum for mid-low rectal carcinoma significantly reduced incidence of vE-AL compared with non-vE-AL (p = 0.042). Patients with vE-AL was associated with presence of peritonitis (p = 0.031), the latter significantly correlated with increased re-operation rate (p = 6.8E-13). Besides, patients with vE-AL trended to correlate with increased re-operation rate after leakage (p = 0.088). In concludsion, vE-AL occurring within 5 post-operative days represents a severe subtype associated with general peritonitis and need of relaparatomy.

Anastomotic leakage (AL) is still one of the most serious complications for colorectal surgery. The incidence is reported about 2.8~8.4% as all^1^,^2^, of which 75% occurs for rectal anastomosis resulting in a mortality rate of 1.7~16.4%^1^,^3^. Thus, understanding the nature and management of this disease is of great clinical importance.

Great bodies of evidence has demonstrated risk factors for AL including male sex, high American Society of Anesthesiologists (ASA) fitness grade, emergency surgery and tumor location, of which neo-adjuvant radiotherapy has been widely regarded as most important one for mid-low rectal carcinoma^1^,^3^,^4^,^5^. However, one recent meta-analysis referring to literature from 1980 to 2015 has demonstrated no significant correlation between increased incidence of AL and neoadjuvant therapy^6^. Defunctioning stoma is demonstrated, by a multicenter randomized controlled trial^7^ and large sample retrospective study using propensity score matching method^8^, effective to reduce the incidence and to mitigate the concequence of AL.

Time of AL is important with respect to the severity and management of this complication. Generally, early AL is associated with severe general peritonitis responsible for emergency relaparatomy and increased motality rate^9^,^10^. By contrast, late AL is associated with long lasting pelvic abscess^11^,^12^,^13^,^14^. Definition of late AL is generally regarded as leakage occurred one month after surgery^12^,^13^,^15^ or patient’s discharge^16^ from majorities of literature. This cut-point time makes late AL a rare event^17^ accounting for less than 4% as indicated by a Korean team reporting 290 AL from 10477 colorectal carcinomas^1^. In fact, median time of AL is about 5–6 post-operative days (range: 1–85 days) from several large sample reports^1^,^2^,^4^,^18^ (22–290 AL from 455–10477 colorectal carcinomas). Therefore, redefinition of early AL with proper cut-point is important for precise discrimination and in-time intervention of lethal cases.

In this study, we analyzed 101 consecutive anastomotic leakage (AL) cases following elective resection of colorectal carcinoma from 2002–2006 in our center. We defined AL occurring ≤5 post-operative days as very early AL (vE-AL). We evaluated risk factors associated with vE-AL, presence of general peritonitis and need of relaparatomy after leakage. We demonstrated that reconstruction of post-peritoneum significantly reduced the incidence of vE-AL for mid-low rectal carcinoma (p = 0.042), the latter significantly correlated with presence of general peritonitis (p = 0.031). More percentages of patients with peritonitis received relaparatomy after leakage (p < 0.001). We suggested that 5 post-operative days was optimal cut-point discriminating true early AL cases associated with general peritonitis needing relaparatomy.

## Results

### General Information of the Cohort

Totally, 101 anastomotic leakages happened, with a median age of 59.0 (15–90) years. 82.2% (83/101) of patients were male. 31.7% (32/101) of patients were T0-2 stage and 45.5% (46/101) with nodal metastasis. Preoperative co-morbidities were not very common including: mellitus diabetes 16.8% (17/101), anemia 7.9%(8/101) and hypo-proteinemia 2.0% (2/101). 69.3% (70/101) of cases were mid-low rectal carcinomas, of which 14 patients received neo-adjuvant chemo-radiotherapy (neo-CRT). 81 open surgeries and 20 laparoscopy assisted surgeries were carried out, 94.1% (95/101) of which was curative. Several techniques were used to reduce risk and severity of leakage, according to clinical experience, including reinforcement of anastomoses by manual sewing (33/101, 32.7%), reconstruction of post-peritoneum (57/70, 81.4%, mid-low rectum) and protective stoma (14/70, 20%, mid-low rectum).

**Figure 1:**
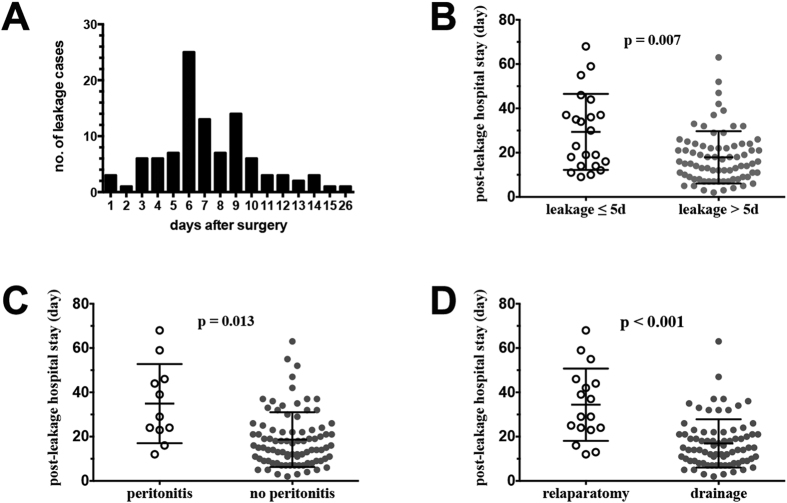
(**A**) Time distribution of 101 anastomotic leakages. **(B–D)** Difference of post-leakage hospital stay between subgroups according to anastomotic leakage time **(B)** presence of peritonitis **(C)** and choices of treatment **(D)**.

Median anastomotic leakage time was 7.0 post-operative days. 23.8% (23/101) of anastomotic leakage occurred within 5 post-operative days (Fig. 1A). Median post-leakage stay and total hospital stay was 18.0 and 29.0 days, respectively. General peritonitis happened in 13 cases and caused severe clinical symptoms. 21 patients received relaparotomy and diverting stoma, of which 2 cases were delayed relaparatomy after failure of conservative therapy. 68 leakages healed before discharge, 29 patients went back with stoma, and 4 patients died of this complication. Details were shown in supplementary Table 1.

### Factors Associated with Very Early Anastomotic Leakage

We evaluated clinicopathologic factors associated with very early anastomotic leakage (vE-AL). As was shown in Table 1, reconstruction of post-peritoneum was the only factor significantly associated with reduced incidence of vE-AL (11/57 vs 6/13, p = 0.042) for mid-low rectal carcinoma. Besides, patients receiving neo-adjuvant chemoradiotherapy for mid-low rectal carcinoma trended to have increased incidence of vE-AL (6/14 vs 11/56, p = 0.070). Anastomotic reinforcement trended to correlate with decreased incidence of vE-AL (4/33 vs 19/68, p = 0.075) for colorectal carcinoma.

### Factors Influencing Peritonitis after Anastomotic Leakage

General peritonitis was the most severe clinical manifestation and leading cause of death. As was shown in Table 2, very early anastomotic leakage (vE-AL, ≤5 post-operative days) was the only significant indicator for general peritonitis (6/23, 26.09% vs 7/78, 8.97%; p = 0.031). Howerer, pre-operative conditions (gender, age, diabetes, anemia, hypo-proteinemia and neo-adjuvant CRT) as well as surgical factors (tumor location, open vs laparoscopy, curative vs palliative) did not significantly affected the incidence of peritonitis. Moreover, some techniques including reinforcement of anastomosis, reconstruction of post-peritoneum and protective soma, which were generally regarded as protective factor to reduce the incidence and severity of anastomotic leakage, did not prevent the presence of peritonitis after leakage.

### Factors Associated with Necessity of Relaparatomy after Anastomotic Leakage

As was shown in Table 3, presence of general peritonitis was the only significant indicator for necessity of relaparotomy after anastomotic leakage (12/13 vs 7/86, p = 6.8E-13). Besides, patients with vE-AL (7/22 vs 12/77, p = 0.088) or diabetes (6/19 vs 11/80, p = 0.064) trended to correlate with increased relaparatomy rate after leakage. However, other perioperative clinicopathologic factors had no significant relationship with relaparatomy.

### Factors Associated with Post-leakage Hospital Stay and Death

We compared post-leakage hospital stay between subgroups according to anastomotic leakage time, presence of peritonitis and treatment modalities. Our results showed that vE-AL (mean: 29.4 vs 17.9 days, p = 0.007, Fig. 1B) and presence of peritonitis (mean: 34.9 vs 18.7 days, p = 0.013, Fig. 1C) significantly correlated with prolonged post-leakage hospital stay. Surprisingly, patients receiving relaparatomy stayed more hospital days after leakage compared with those receiving conservative drainage (mean: 34.4 vs 16.9 days, p = 4.6E-4, Fig. 1D).

Unfortunately, 4 patients died because of this complication. Strikingly, leakage associated death did not mainly occur in high-risk patients including patients with very early leakage (1/4), peritonitis (1/4) or lower rectal carcinoma (1/4). Three leakages of colon anastomoses occurred 7 days after surgery and did not show severe symptoms of peritonitis, which resulted in the loss of opportunity for relaparatomy.

## Discussion

Anastomotic leakage (AL) was one of the most important complications after surgery of colorectal carcinoma, which was responsible for prolonged hospital stay, delayed post-operative chemoradiotherapy, increased re-operation and motality rate^19^. Patients with high risk of AL would receive protective stoma during first operation, which was planned to be reversed within 3–6 months. However, these temporary stomas would become permanent due to lower rectal anastomotic stricture following radiotherapy. This would largely reduce quality of life, especially for younger patients. Therefore, understanding the mechanism of AL is of great clinical significance.

Early anastomotic leakage, defined as AL occurring within 30 post-operative days^12^,^16^, represents a unique entity requiring more aggressive intervention^12^,^13^ and causing elevated motality rate compared with late AL. However, since majorities of AL occurred 5–10 days after surgery, cut-point of 30 post-operative days makes late AL rarity^17^ accounting for less than 4 percent of AL cases as indicated by a Korean team reporting 290 AL from 10477 colorectal carcinomas^1^. Therefore, redefinition of early anastomotic leakage with optimal cut-point time may be helpful to distinguish truly lethal cases needing urgent intervention.

In the present study, we demonstrated that very early AL (vE-AL, ≤5 post-operative days) was significantly associated with presence of general peritonitis (p = 0.031, Table 2) and trended to correlate with increased urgent relaparatomy rate (p = 0.088, Table 3). This was in accordance with great bodies of evidence demonstrating early AL as severe and lethal complication. Floodeen H *et al*.^9^ reported that early symptomatic AL (diagnosed during initial hospital stay, average 8 post-operative days) performed worse than late AL (diagnosed after discharge, average 22 post-operative days) with regard to oral intake, bowel activity and initial hospital stay. Early AL within 30 days after surgery was associated with increased proportions of patients needing relaparatomy (78.8–89% vs 44–55.4%)^12^,^13^. Several factors may contribute to this discrepancy. First, early AL was associated with worse local inflammation, determined by elevated levels of inflammatory cytokines (TNF-α, IL-6, IL-12)^20^,^21^,^22^ from peritoneal fluid as early as the first post-operative day, as well as systemic inflammation burst in terms of elevated expression of circulating procalcitonin (PCT) and C-reactive protein^23^. This inflammatory stress deteriorates the pre-existed negative-nitrigen nutritional status of the patient, whose oral intake and bowel function has not completely recovered, leading to delayed healing of AL. In turn, worsening of AL accelerates nutritional consumption due to restriction of oral intake. These two processes exacerbate each other in a loop resulting in disease progression. Second, post-operative abdominal adhension is critical for the re-formation of post-peritoneum leading to localization of pelvic inflammation. However, in very early AL cases occurring within 5 days after surgery, abdominal adhension has not substantially formatted, which enables penetration and dissemination of pelvic leaking feces into abdominal cavity leading to severe general peritonitis^11^. This can partly explain why reconstruction of post-peritoneum does not reduce the severity of vE-AL in terms of peritonitis (p = 0.473, Table 2). Therefore, vE-AL might be life-threatening, especially for those atypical elderly cases following chemoradiotherapy^24^. About 1/3 of AL cases might present without symptoms^18^. In this study, one elderly patient undergoing ultra-low rectal anastomosis 5 days after oral capecitabine chemotherapy died of very early AL at the first post-operative day. This patient behaved, without evident feces drainage or signs of peritonitis, as fever and severe agranulocytosis which rapidly progressed to septic shock within 24 hours before surgical intervention could be applied. Indeed, as the fact that majorities of AL happened at least one week after surgery, very early AL of elderly patients with bluntness physical reaction could be easily underestimated. Delayed diagnosis or persistent conservative therapy was reasonably dangerous.

General peritonitis is one of the most severe outcome for AL. Lim SB^25^ analyzed 142 AL from 2510 consecutive colorectal carcinomas and found that generalized peritonitis (type I AL) was the most common type (44.7%). Generalized peritonitis was associated with increased re-operation rate, as high as 100% in Rickert A’s report (67 AL from 1731 colorectal anastomosis) which demonstrated peritonitis as one of the only two reasons for not preserving the anastomosis^26^. Infected peritonitis also correlated with decreased short term and long term survival of AL patients according to the report by Salvans S *et al*.^27^ that infected peritoneal fluid enhanced cell migration and invasion of colorectal MDA-MB-231/SW620 cell lines *in vitro*. Two-year disease-free survival of patients with peritoneal infection was significantly lower (77.6% vs 90.6%). In our study, leakage-associated peritonitis was the only significant indicator of relaparatomy (p = 6.8E-13, Table 3). It is noteworthy whether re-operation is attributed to life-threatening peritonitis or the fasioned choice by the surgeon is still unknown. Besides, little evidence has been concluded about short-term outcome of leakage-associated peritonitis according to different choices of conservative or surgical interventions^28^.

In conclusion, we analyzed 101 consecutive colorectal anastomotic leakages and defined AL occurring ≤5 post-operative days as very early anastomotic leakage (vE-AL). We found that reconstruction of post-peritoneum for mid-low rectal carcinoma significantly reduced incidence of vE-AL compared with late AL (p = 0.042). Patients with vE-AL was associated with presence of peritonitis (p = 0.031), the latter significantly correlated with increased re-operation rate (p = 6.8E-13). We suggested that 5 post-operative days was optimal cut-point time discriminating truly life-threatening early AL needing urgent relaparatomy.

## Methods

### Patients

101 consecutive patients with symptomatic anastomotic leakage following elective surgery for colorectal carcinoma in Cancer Center of Fudan University (Shanghai, China) between January 2013 and Dec 2015 were enrolled in this study. Medical records were retrospectively reviewed and follow up was carried out at least 3 moths after discharge. This study was approved by the Research Ethics Committee of Cancer Hospital, Fudan University. Collection of patient tissues and follow-up data was in accordance with guideline for collection of human tissue and follow-up data of Cancer Hospital, Fudan University. Informed consent was obtained from all participants. Detialed information of patient chareteristics was shown in Supplementary Table 1.

### Definition of Anastomotic Leakage

Anastomotic leakage was defined as a communication between the intra- and extraluminal compartments owing to a defect of the integrity of the intestinal wall at the anastomosis (proposed by the **International Study Group of Rectal Cancer**, Surgery 2010;147:339-51). Detailed criteria were as follows: (1) apparent discharge of gas/pus/feces from abdominal or pelvic drainage tube; (2) anastomotic defect confirmed by proctoscopy, CT scan using contrast medium or rectal examination (only for lower rectal anastomosis); (3) confirmed during relaparatomy.

### Peri-operative Preparation and Surgical Procedure

Bowel preparation, peri-operative administration of antibiotics, enteral nutrition (EN) and/or parenteral nutrition (PN) was comparable for the whole cohort. Oral polyethylene glycol solution was routinely taken the last night before surgery unless patients had symptoms of obstruction or perforation. Human albumin supplementation or blood transfusion was adopted if necessary. Standard **C**omplete **M**esocolic **E**xcision (CME) or **T**otal **M**esocolic **E**xcision (TME) was carried out as described elsewhere. The anastomosis was carried out using stapler for all the patients. Manual reinforcement sewing was performed when anastomosis was above the peritoneal reflextion.

### Statistical analysis

Statistical analysis was performed using SPSS version 22. Comparison of subgroup differences was determined using chi-square test or Mann-whitney U test, as appropriate. Two sided p value of less than 0.05 was considered as statistically significant.

## Additional Information

**How to cite this article**: Li, Y.-W. *et al*. Very Early Colorectal Anastomotic Leakage within 5 Post-operative Days: a More Severe Subtype Needs Relaparatomy. *Sci. Rep.* **7**, 39936; doi: 10.1038/srep39936 (2017).

**Publisher's note:** Springer Nature remains neutral with regard to jurisdictional claims in published maps and institutional affiliations.

## Supplementary Material

Supplementary Table 1

## Figures and Tables

**Table 1:** Risk Factors of very-Early Anastomostic Leakage.

		Anastomotic leakage			
Variables		≤5d	>5d	χ^2^	p value
**Gender**	Male	20	63		
	Female	3	15	0.464	0.496
**Age (year)**	<70	20	65		
	≥70	3	13	0.175	0.676
**Diabetes**	Yes	5	12		
	No	18	66	0.512	0.474
**Anemia**	Yes	2	6		
	No	21	72	0.025	0.876
**Hypo-proteinemia**	Yes	0	2		
	No	23	76	0.602	0.438
**T stage**	0–2	8	24		
	3–4	15	54	0.132	0.716
**N stage**	0	15	40		
	1–2	8	38	1.391	0.238
*neo-CRT	Yes	6	8		
	No	11	45	3.282	0.070
**Tumor location**	Colon/high rectum	6	25		
	Mid-low rectum	17	53	0.297	0.586
**Laparascopy**	Yes	7	13		
	No	16	65	2.120	0.145
**Curative resection**	Yes	22	73		
	No	1	5	0.135	0.713
**Reinforce of anastomosis**	Yes	4	29		
	No	19	49	3.162	0.075
*Protective stoma	Yes	3	14		
	No	11	42	0.078	0.780
*Reconstruction of post-peritoneum	Yes	11	46		
	No	6	7	4.152	0.042

*for mid-low rectal carcinoma only (n = 70).

**Table 2:** Risk Factors of Leakage-associated General Peritonitis.

		Peritonitis			
Variables		Yes	No	χ^2^	p value
**Gender**	Male	11	72		
	Female	2	16	0.061	0.806
**Age (year)**	<70	10	75		
	≥70	3	13	0.586	0.444
**Diabetes**	Yes	4	13		
	No	9	75	2.07	0.150
**Anemia**	Yes	0	8		
	No	13	80	1.283	0.257
**Hypo-proteinemia**	Yes	1	1		
	No	12	87	2.508	0.113
**T stage**	0–2	5	27		
	3–4	8	61	0.317	0.574
**N stage**	0	8	47		
	1–2	5	41	0.302	0.583
*neo-CRT	Yes	2	5		
	No	12	51	0.357	0.550
**Tumor location**	Colon/high rectum	6	25		
	Mid-low rectum	7	63	1.677	0.195
**Laparascopy**	No	10	71		
	Yes	3	17	0.101	0.751
**Curative resection**	Yes	11	84		
	No	2	4	2.382	0.123
**Reinforce of anastomosis**	Yes	4	29		
	No	9	59	0.025	0.875
*Reconstruction of post-peritoneum	Yes	5	52		
	No	2	11	0.514	0.473
*Protective stoma	Yes	1	13		
	No	6	50	0.159	0.690
**Leakage time (days after surgery)**	≤5	6	17		
	>5	7	71	4.638	0.031

*for mid-low rectal carcinoma only (n = 70).

**Table 3:** Risk Factors Associated with Relaparotomy.

		Treatment modality**			
Variables		drainage	relaparotomy	χ^2^	p value
**Gender**	Male	66	15		
	Female	14	4	0.130	0.718
**Age (year)**	<70	70	14		
	≥70	10	5	2.280	0.131
**Diabetes**	Yes	13	6		
	No	69	11	3.431	0.064
**Anemia**	Yes	7	1		
	No	73	18	0.251	0.616
**Hypo-proteinemia**	Yes	2	0		
	No	78	19	0.485	0.486
**T stage**	0–2	24	8		
	3–4	56	11	1.028	0.311
**N stage**	0	41	12		
	1–2	39	7	0.875	0.350
**neo-CRT**	Yes	12	2		
	No	44	10	0.137	0.711
**Tumor location**	Colon/high rectum	24	7		
	Mid-low rectum	56	12	0.334	0.563
**Laparascopy**	Yes	16	4		
	No	64	15	0.011	0.918
**Curative resection**	Yes	75	18		
	No	5	1	0.026	0.871
**Reinforce of anastomosis**	Yes	27	6		
	No	53	13	0.033	0.857
**Reconstruction of post-peritoneum**	Yes	47	9		
	No	9	3	0.542	0.462
**Protective stoma**	Yes	12	2		
	No	44	10	0.137	0.711
**Leakage time (days after surgery)**	≤5	15	7		
	>5	65	12	2.908	0.088
**Peritonitis**	Yes	1	12		
	No	79	7	51.586	6.8E-13

*for mid-low rectal carcinoma only (n = 70). **two cases receiving delayed relaparatomy due to failure of conservative therapy were excluded.
