# Supplementary material for: Very Early Colorectal Anastomotic Leakage within 5 Post-operative Days: a More Severe Subtype Needs Relaparatomy
Source: Sci Rep. 2017 Jan 13;7:39936. doi: 10.1038/srep39936 (PMC5233968; doi:10.1038/srep39936)
Supplement: Supplementary Table 1 [file srep39936-s1.doc]

**Very Early Colorectal Anastomotic Leakage within 5 Post-operative Days: a More Severe Subtype Needs Relaparatomy**

Yi-Wei Li^1^, Peng Lian^1*^, Ben Huang^1^, Hong-Tu Zheng^1^, Ming-He Wang^1^, Wei-Lie Gu^1^, Xin-Xiang Li^1^, Ye Xu^1^, San-Jun Cai^1*^.

**Supplementary Table 1.** Patient Characteristics of the Cohort

| **Variables** | **Classification** | **No** |
| --- | --- | --- |
| Gender | Male | 83 |
|  | Female | 18 |
| Age (year) | <70 | 85 |
|  | ≥70 | 16 |
| Diabetes | Yes | 17 |
|  | No | 84 |
| Anemia | Yes | 8 |
|  | No | 93 |
| Hypo-proteinemia | Yes | 2 |
|  | No | 99 |
| *Neo-CRT | Yes | 14 |
|  | No | 56 |
| Tumor location | Colon/high rectum | 31 |
|  | Mid-low rectum | 70 |
| Laparascopy | Yes | 20 |
|  | No | 81 |
| Curative resection | Yes | 95 |
|  | No | 6 |
| Tumor stage | T0 | 5 |
|  | T1 | 6 |
|  | T2 | 21 |
|  | T3 | 62 |
|  | T4 | 7 |
| Nodal stage | N0 | 55 |
|  | N1 | 31 |
|  | N2 | 15 |
| Anastomosis reinforcement | Yes  No | 33  68 |
| *Post-peritoneum close | Yes  No | 57  13 |
| *Protective stoma | Yes  No | 14  56 |
| Leakage time  (days after surgery) | ≤5  > 5 | 23  78 |
| General peritonitis | Yes  No | 13  88 |
| Treatment | Drainage  Relaparatomy | 80  19 |
|  | Delayed relaparatomy** | 2 |
| Results | Normal discharge  Discharge with stoma  Death | 68  29  4 |
| * for mid-low rectal carcinoma only (n = 70)  ** delayed relaparatomy after failure of conservative therapy | | |
|  |  |  |
